# Elevated C-reactive protein and D-dimer to predict venous thromboembolism in patients with bladder cancer

**DOI:** 10.3389/fimmu.2025.1652139

**Published:** 2025-08-13

**Authors:** Bo Chen, Tonghe Zhang, Yisong Wang, Zhaoyang Li, Haoyu Liu, Zhan Jiang, Huitang Yang, Yandong Cai, Guoju Fan, Kaiqiang Wang, Hongwei Zhang, Hailong Hu, Yankui Li

**Affiliations:** ^1^ Department of Vascular Surgery, The Second Hospital of Tianjin Medical University, Tianjin, China; ^2^ Center for Cardiovascular Diseases, The Second Hospital of Tianjin Medical University, Tianjin, China; ^3^ Clinical Medical College, Hebei University, Baoding, Hebei, China; ^4^ Department of Urology, The Second Hospital of Tianjin Medical University, Tianjin, China; ^5^ Tianjin Key Laboratory of Urology, Tianjin Institute of Urology, Tianjin, China

**Keywords:** venous thromboembolism, C-reactive protein, d-dimer, bladder cancer, inflammation, immune system

## Abstract

**Objective:**

This study evaluated C-reactive protein (CRP) in hospitalized patients with bladder cancer (BC) and explored the predictive value of CRP for venous thromboembolism (VTE), combining CRP and D-dimer (D-D) levels to improve the ability to predict the risk of VTE in BC patients, thereby better guiding clinical prevention and treatment of this disease.

**Methods:**

Clinical data from 4,438 patients with BC admitted between January 2015 and December 2020 were reviewed. After screening, 2,164 patients remained.52 VTE cases were identified, and 104 matched controls were selected (1:2 ratio). Conditional logistic regression, receiver operating characteristic (ROC) curve analysis, stratified analysis, and interaction tests were conducted to assess predictive performance and control for confounding bias.

**Results:**

Conditional logistic regression analysis indicated that elevated CRP and D-D levels were associated with higher risk of VTE in hospitalized patients with BC. Moreover, the areas under the ROC curves were 0.734 for CRP, 0.817 for D-D, and 0.865 for the combined model, indicating that the combined model offers superior predictive performance. Stratified and interaction analyses further revealed that the predictive value of CRP and D-D levels was influenced by the infection status.

**Conclusion:**

Elevated CRP and D-D levels may be potential indicators of VTE in BC patients. Their combined use improves predictive accuracy, and their predictive value may be better in non-infected patients.

## 1 Introduction

Venous thromboembolism (VTE) is a serious clinical condition that includes deep vein thrombosis (DVT) and pulmonary embolism (PE) (1, 2). In cancer patients, the risk of VTE ranges from 3% to 5% in early-stage disease and up to 30% in advanced-stage or metastatic cancer (3, 4). The overall incidence of VTE in patients with bladder cancer (BC) ranges from 0.4% to 4.7% (5, 6). VTE is not only a disorder of coagulation but also a complex immunoinflammatory process (7–9). In patients with BC, the development of VTE involves multiple interrelated factors, such as tumor biology, treatment modalities, and patient-specific characteristics (10–12). Without early identification and prevention, severe VTE events, such as pulmonary embolism, can lead to sudden death or disrupt the course of cancer treatment (13, 14). Therefore, early screening of high-risk individuals and timely initiation of anticoagulant prophylaxis are crucial to reduce VTE incidence, improve patients’ quality of life, and enhance clinical outcomes, making them vital components of comprehensive cancer management (15, 16).

VTE is increasingly recognized as a classic example of immunothrombosis, in which systemic inflammation significantly elevates thrombotic risk (17, 18). C-reactive protein (CRP)—a highly conserved member of the pentraxin family—is widely used as a biomarker of infection and inflammation in clinical practice and is typically measured using either traditional or high-sensitivity CRP assays (19–21). CRP possesses both proinflammatory and prothrombotic properties and plays a central role in the pathogenesis of arterial and venous thrombosis (20, 22). D-dimer (D-D)—a soluble fibrin degradation product generated through plasmin-mediated fibrinolysis of cross-linked fibrin—is a well-established biomarker of coagulation activation and secondary fibrinolysis (23, 24). Elevated plasma D-D levels have frequently been associated with the pathophysiology of VTE (25, 26). Research suggests that CRP and D-D may be potential prognostic biomarkers for patients with cancer and for the recurrence of VTE after discontinuation of anticoagulant therapy in cancer-related thrombosis (27–29).

An individualized medical approach that integrates immunological and coagulation markers may offer a superior strategy for preventing and managing VTE in patients with BC (30). However, the current guideline-based evidence for VTE risk assessment in patients with BC remains limited. Therefore, this study aimed to explore the predictive value of CRP and D-D levels, individually and in combination, for VTE in patients with BC. We hypothesize that evaluating these markers in combination will improve the accuracy of early-risk identification and screening, reduce the incidence of VTE and related mortality, and ultimately improve clinical outcomes for patients.

## 2 Materials and methods

### 2.1 Patient source

**Figure 1:**
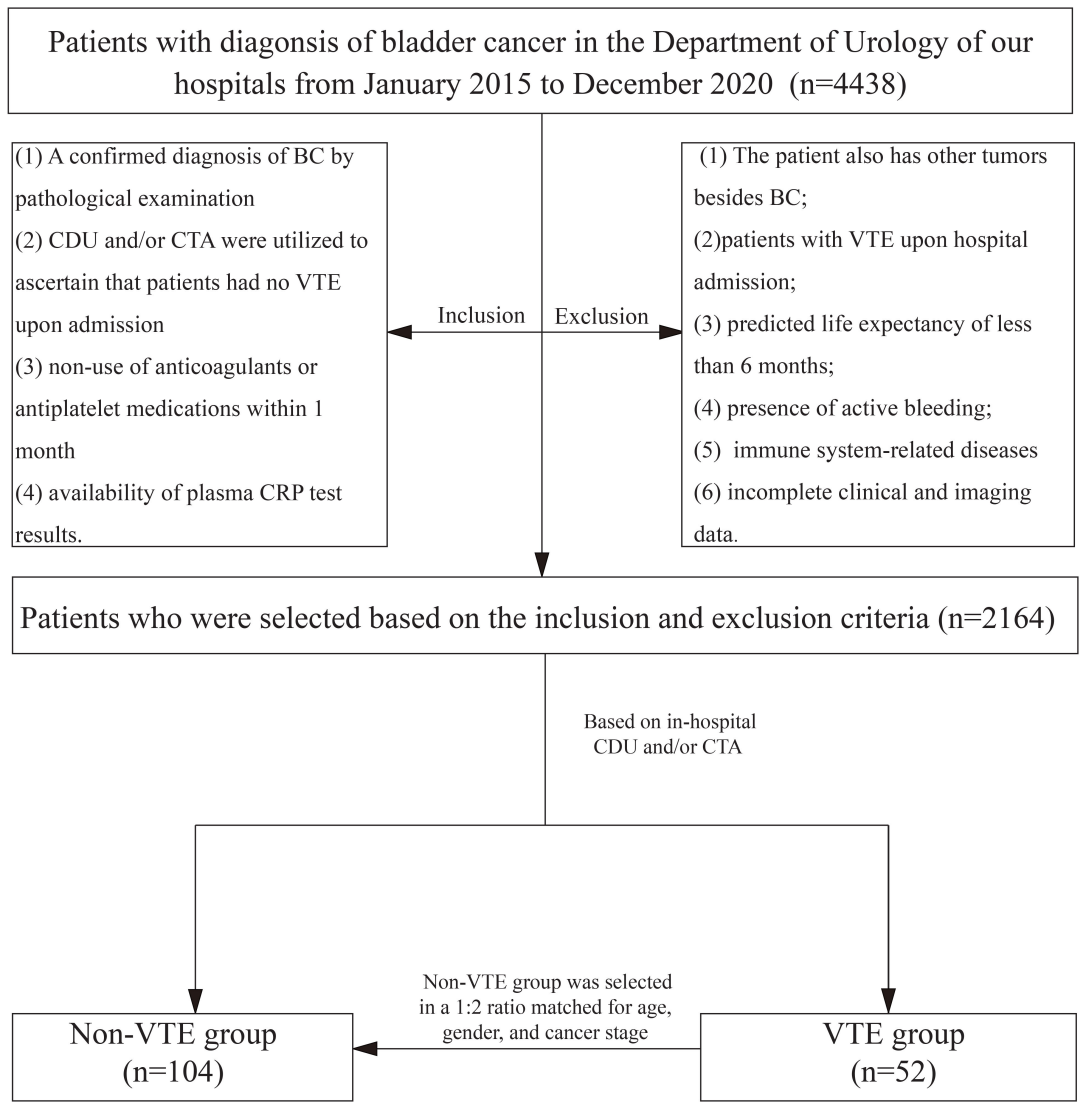
Flowchart for the enrolled patients.

This study initially screened 4,438 patients diagnosed with BC who were admitted to the Department of Urology at our hospital between January 2015 and December 2020. After applying the inclusion and exclusion criteria, 2,164 patients were eligible for further analysis. Of these, 52 patients were selected for the VTE group based on in-hospital imaging results, and patients without VTE (controls) were selected and matched in a 1:2 ratio, considering age, sex, and cancer stage, yielding a final study population of 156 patients (**Figure 1**).

### 2.2 Patient screening criteria

The inclusion criteria were as follows: (1) A confirmed diagnosis of BC by pathological examination, (2) color Doppler ultrasound (CDU) and/or computed tomography angiography (CTA) were utilized to ascertain that patients had no VTE upon admission, (3) non-use of anticoagulants or antiplatelet medications within one month, and (4) availability of plasma CRP test results(immunoturbidimetric method was used for CRP detection in our hospital; CRP reference range: 0–10 mg/L). The exclusion criteria were as follows: (1) The patient had other tumors besides BC, (2) patients with VTE upon hospital admission, (3) predicted life expectancy of less than 6 months, (4) presence of active bleeding, (5) immune system-related diseases, and (6) incomplete clinical and imaging data.

### 2.3 Data collection

Patients’ clinical characteristics, including, but not limited to, age, sex, body mass index (BMI), cancer stage, history of lifestyle or related disease (alcohol consumption, smoking, diabetes mellitus, and hypertension), infection, CRP level, D-D level, surgery received, and medications administered, were extracted from the electronic medical record system of our hospital. Patients identified as high risk (Caprini risk score ≥ 5) routinely underwent lower limb venous CDU screening. For those presenting with chest pain, cough, or dyspnea in addition to lower limb DVT, pulmonary CTA was performed to assess for PE.

### 2.4 Statistical analysis

R software (version 4.4.0; R Foundation for Statistical Computing, Vienna, Austria) was used for statistical analysis. Continuous data are shown as the mean ± standard deviation (SD). Student’s t-test was used for normally distributed data and the Mann–Whitney U test for non-normally distributed data. All categorical data are shown as frequencies and rates, and the chi-square (χ²) test was used to assess comparisons between groups. Conditional logistic regression analysis was applied, and receiver operating characteristic (ROC) curves were constructed to evaluate the discriminatory ability of CRP, D-D, and their combination in predicting VTE. In addition, stratified analysis and interaction testing were performed to assess whether infection status modified the associations between CRP, D-D, and VTE risk.

## 3 Results

The baseline characteristics of the enrolled patients are summarized in **Table 1**, along with comparative outcomes between the VTE and non-VTE groups. A total of 156 patients were included in this study: 52 patients with VTE and 104 without VTE. Among the 52 patients with VTE, 3 had PE (with concurrent DVT), and all 52 had DVT: 43 patients had below-knee venous thrombosis (including posterior tibial vein, anterior tibial vein, peroneal vein, and intermuscular vein), 2 had popliteal vein thrombosis, and 7 had femoral vein thrombosis. The VTE and non-VTE groups were similar regarding baseline characteristics, including age, sex, BMI, cancer stage, and history of smoking, alcohol consumption, hypertension, and surgeries and medications received, indicating that the matching method applied was appropriate (**Table 1**). However, after matching, statistically significant differences remained in the history of diabetes mellitus, CRP level, and D-D level between the VTE and non-VTE groups (p < 0.05).

**Table 1:** Baseline characteristics and different treatments of enrolled patients.

Characteristics	Non-VTE group (n=104)	VTE group (n=52)	p
Age, Mean ± SD	71.42 ± 7.67	71.50 ± 7.44	0.952
Sex, n (%)			
male	82 (78.85%)	41 (78.85%)	1.000
female	22 (21.15%)	11 (21.15%)	
BMI, Mean ± SD	23.51 ± 2.82	24.02 ± 2.58	0.259
Smoking history, n (%)	44 (42.31%)	23 (44.23%)	0.954
Alcohol consumption, n (%)	21 (20.19%)	16 (30.77%)	0.206
Hypertension, n (%)	46 (44.23%)	23 (44.23%)	1.000
Diabetes mellitus, n (%)	15 (14.425%)	18 (34.62%)	0.007^**^
Infection, n (%)	31 (29.82%)	14 (26.92%)	0.851
CRP, Mean ± SD	19.37 ± 30.14	81.58 ± 176.92	0.015^*^
D-D, Mean ± SD	0.8 ± 1	4.64 ± 11.34	0.018^*^
Surgery, n (%)			
TURBT	51 (49.08%)	21 (40.38%)	0.577
RC	26 (25.00%)	16 (30.77%)	
Medications, n (%)			
Chemotherapy alone	59 (56.73%)	26 (50.00%)	0.075
Chemotherapy + Immunotherapy	13 (12.50%)	14 (26.92%)	
Cancer stages, n (%)			
I	26 (25.00%)	13 (25.00%)	1.000
II	48 (46.15%)	24 (46.15%)	
III	24 (23.08%)	12 (23.08%)	
IV	6 (5.77%)	3 (5.77%)	

*p<0.05 **p<0.01 BMI, body mass index; CRP, C-reactive protein; D-D, D-dimer; TURBT, transurethral resection of bladder tumor; RC, radical cystectomy; VTE, venous thromboembolism.

To explore the potential of CRP and D-D as predictive markers of VTE in hospitalized patients with BC, we performed a conditional logistic regression analysis. The clinical data of patients with BC were included in the conditional logistic regression analysis to identify predictors of in-hospital VTE. Age, sex, BMI, cancer stage, alcohol consumption, smoking history, diabetic status, hypertension history, infection status, CRP level, D-D level, surgery received, and medications administered were input as independent variables in the conditional logistic analysis, with the in-hospital VTE incidence as the dependent variable. Elevated CRP and D-D levels were independently associated with an increased risk of VTE (p < 0.05). These results suggest that CRP and D-D levels may serve as valuable predictive markers of VTE in patients with BC (**Table 2**).

**Table 2:** Conditional logistic regression analysis for VTE in patients with bladder cancer.

Variable	Beta.	OR	95% CI	p
CRP	0.023	1.024	0.009–0.038	0.002^**^
D-D	0.65	1.915	0.287–1.013	<0.001^**^

**p<0.01 CRP, C-reactive protein; D-D, D-dimer; OR, odds ratio; CI, confidence interval; VTE, venous thromboembolism.

**Figure 2:**
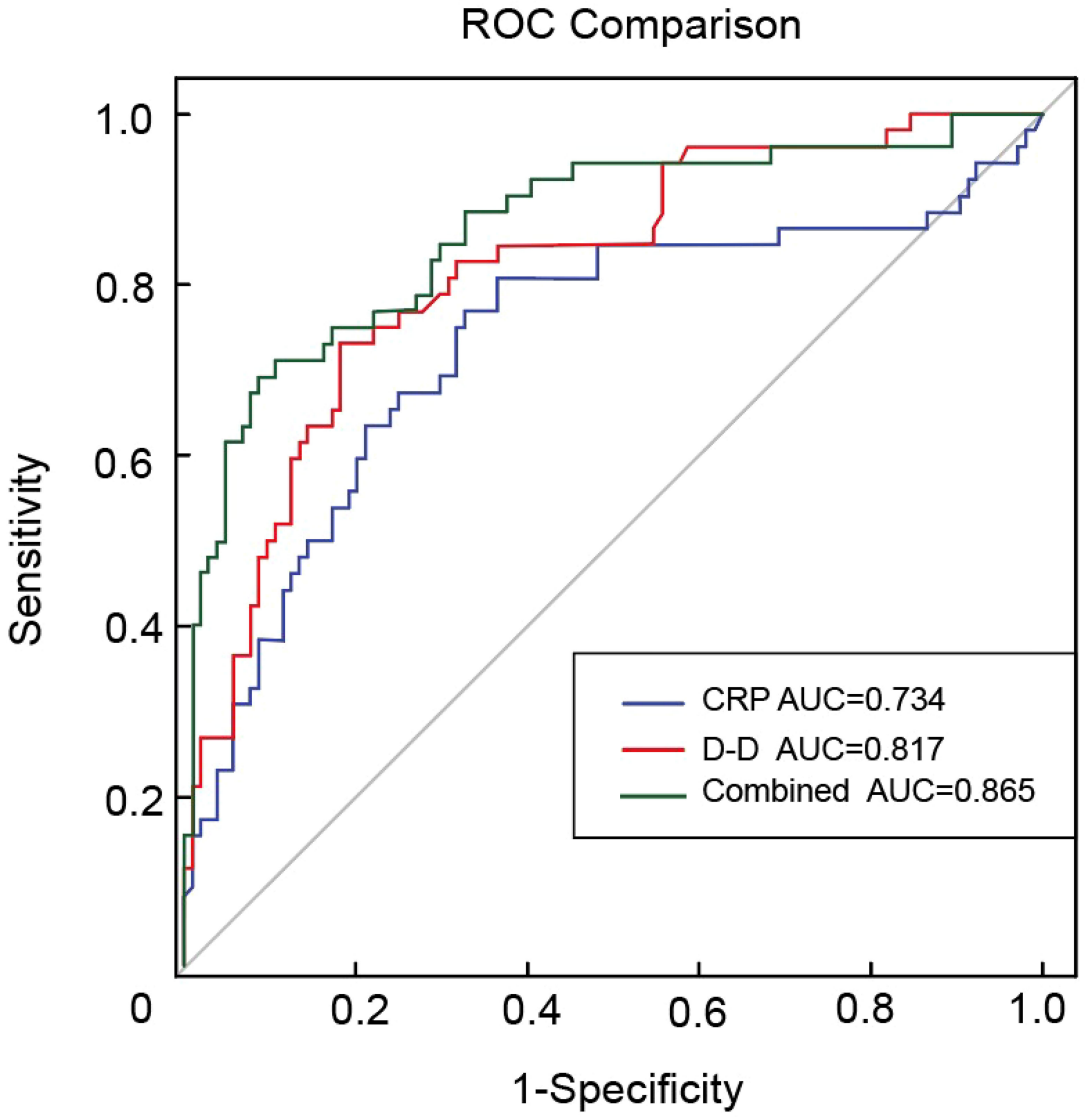
ROC curves for CRP, D-D, and their combination in predicting VTE in bladder cancer. ROC, receiver operating characteristic; CRP, C-reactive protein; D-D, D-dimer; VTE, venous thromboembolism.

ROC curves were constructed to evaluate the predictive ability of CRP levels, D-D levels, and their combination for VTE in patients with BC. The area under the curve (AUC) values were 0.734 for CRP, 0.817 for D-D, and 0.865 for the combined model, indicating that the combined index had the highest discriminatory ability. These results suggest that integrating CRP and D-D levels may improve the predictive performance of VTE risk stratification in this population (**Figure 2**).

To minimize the potential confounding effect of infection, a stratified analysis was conducted based on the infection status. The results indicated that both CRP and D-D levels were significantly associated with VTE in the non-infected group (p < 0.05), whereas no such associations were observed in the infected group (p > 0.05). These findings support that CRP and D-D plasma levels may serve as reliable predictive markers for VTE in patients with BC without infection (**Supplementary Table S1**).

Interaction analysis confirmed the modifying effect of infection on the predictive value of the CRP level. A significant interaction was observed between CRP levels and infection (p = 0.003), indicating that the infection status may attenuate the predictive ability of the CRP level for VTE. In contrast, no significant interaction was found between D-D and infection (p = 0.313), suggesting that the D-D level remains a relatively stable predictor, regardless of infection status (**Supplementary Table S2**).

## 4 Discussion

VTE is not only a manifestation of abnormal coagulation but also a classic process of immunothrombosis, involving complex interactions between the inflammatory and coagulation systems (31, 32). In patients with malignancies, such as BC, the risk of VTE is significantly increased due to tumor-induced hypercoagulability, prolonged immobilization, surgical intervention, and chemotherapy (33, 34). Identifying biomarkers that simultaneously reflect inflammation and coagulation status is crucial for early prediction and prevention of VTE in high-risk populations.

CRP, an acute-phase reactant, is a sensitive marker of systemic inflammation that promotes thrombosis by enhancing endothelial dysfunction, activating coagulation pathways, and impairing fibrinolysis (35, 36). In the present study, elevated CRP level in the absence of infection was statistically significantly associated with VTE in hospitalized patients with BC (p < 0.01), suggesting its potential utility as a predictive marker. This finding is consistent with that of a previous report highlighting the predictive role of CRP in thrombogenesis (36). CRP measurement is cost-effective, widely available, and easy to perform in clinical settings (37, 38). However, in the current study, stratified analysis by infection status showed that infection may attenuate the specificity of CRP (p > 0.05), and an interaction test further confirmed the significant modifying effect of infection on the CRP–VTE association (p < 0.05). These findings highlight the importance of considering background inflammatory conditions when interpreting CRP levels in clinical practice.

This study is the first to assess the risk of VTE in patients with BC using CRP and D-D levels combined, addressing not only coagulation dysfunction but also the immunoinflammatory aspects of thrombosis. Our study revealed a statistically significant association between elevated D-D levels and VTE incidence, consistent with the findings of previous studies (39, 40). ROC curve analysis revealed AUCs of 0.734 for CRP, 0.817 for D-D, and 0.865 for the combined model. The combination of CRP and D-D significantly improved the predictive performance of the model compared to either marker alone, suggesting that integrating inflammatory and coagulation markers may enhance the clinical utility of risk assessment models. This combined approach may improve the efficiency of VTE screening in patients with BC. This further supports the development of more precise preventive strategies by highlighting the importance of managing thromboinflammatory responses in parallel with anticoagulation therapy, with the aim of lowering the incidence and mortality of VTE.

These findings suggest that CRP and D-D levels are promising biomarkers for individualized VTE risk assessment in patients with BC. However, there are some limitations to this study. First, the retrospective design did not fully exclude patient heterogeneity in reasons for hospitalization. We plan to address this issue further in future research by collecting data from a large number of patients and conducting more rigorous stratified analyses. Second, due to the retrospective nature of the study and limited availability of clinical data, time series data was unavailable for most patients. To enhance the reliability and clinical relevance of these findings, future prospective multicenter studies incorporating dynamic biomarker monitoring are needed.

## 5 Conclusion

This study demonstrated that elevated CRP and D-D levels were statistically associated with an increased risk of VTE in hospitalized patients with BC. Our findings suggest that integrating CRP and D-D levels may provide a useful strategy for the early identification and risk stratification of VTE in BC, particularly in patients without infection.

## Data Availability

The original contributions presented in the study are included in the article/**Supplementary Material**. Further inquiries can be directed to the corresponding author/s.
