# Supplementary material for: Elevated C-reactive protein and D-dimer to predict venous thromboembolism in patients with bladder cancer
Source: Front Immunol. 2025 Aug 13;16:1652139. doi: 10.3389/fimmu.2025.1652139 (PMC12380552; doi:10.3389/fimmu.2025.1652139)
Supplement: Supplementary file 1 [file Table1.docx]

Supplementary Material

| **Supplementary Table S1.** Stratified analysis based on infection status | | | | |
| --- | --- | --- | --- | --- |
|  | **Variables** | **OR** | **95% CI** | **p** |
| Non-infection  (n=111) | CRP | 1.039 | 1.014–1.064 | 0.002^**^ |
|  | D-D | 2.507 | 1.227–5.122 | 0.012^*^ |
| Infection  (n=45) | CRP | 1.007 | 0.980–1.034 | 0.630 |
|  | D-D | 1.089 | 0.293–0.614 | 0.770 |
| *p<0.05 **p<0.01 | | | | |

| **Supplementary Table S2.** Interaction analysis between infection and biomarkers in predicting VTE | | | |
| --- | --- | --- | --- |
| **Variables** | **OR** | **95% CI** | **p** |
| CRP | 1.036 | 1.015–1.057 | 0.001^**^ |
| Infection | 3.023 | 0.761–12.009 | 0.116 |
| D-D | 2.215 | 1.282–3.826 | 0.004^**^ |
| Infection × CRP | 0.968 | 0.947–0.989 | 0.003^**^ |
| Infection × D-D | 0.683 | 0.325–1.432 | 0.313 |
| *p<0.05 **p<0.01 | | | |
